# Paternal early life stress exerts intergenerational effects on male C57Bl/6J offspring risk-taking behaviors and predator scent-induced c-Fos expression

**DOI:** 10.1042/NS20220097

**Published:** 2023-04-28

**Authors:** Ulysse M.C.C. Thivisol, Phoebe Ho, Baijia Li, Mari Trompke, Lucas B. Hoffmann, Anthony J. Hannan, Terence Y. Pang

**Affiliations:** 1 The Florey Institute of Neuroscience and Mental Health, Parkville, VIC 3052, Australia; 2 Department of Anatomy and Physiology, University of Melbourne, VIC 3010, Australia; 3 The Florey Department of Neuroscience and Mental Health, University of Melbourne, VIC 3010, Australia

**Keywords:** innate fear, intergenerational inheritance, maternal separation, Paternal stress, predator odor, TMT

## Abstract

Paternal preconceptional health factors, such as exposures to stress, diet and exercise, have been found to significantly influence offspring phenotypes in a range of animal models. Preclinical studies have provided evidence that paternal stress is associated with increased stress responsivity and anxiety-related traits, particularly in male offspring. It was previously reported that a paternal history of maternal separation (MS) led to male offspring (PatMS) displaying reduced cautious behavior during exploration of a novel environment. The neural basis for that absence of behavioral moderation is unclear. Here, we investigated the adaptive behavioral responses of control and PatMS male offspring in the predator odor risk-assessment task (PORT). PatMS mice failed to moderate their behaviors in the presence of a predator odor 2,4,5-trimethylthiazoline (TMT). c-Fos mapping revealed reduced cellular activation in fear-regulating brain regions of PatMS mice, such as in the cingulate cortex, dentate gyrus of the hippocampus and the basolateral amygdala. Expression of the paternally imprinted gene Grb10 (previously identified as a key molecular regulator of risk-taking behavior) was unaltered in PatMS mice. However, other paternal imprinted genes such as Igf2 and PEG3 were differentially expressed in PatMS mice. Overall, our study provides the first evidence of an intergenerational influence of preconceptional paternal stress exposure on offspring brain zunction relevant to risk-taking behavior, which is also independent of Grb10 gene expression.

## Introduction

Risk assessment is an adaptive cognitive mechanism for integrating environmental information to evaluate potential consequences and rewards, in order to optimize behavioral strategies [1]. Impaired risk assessment can lead to higher risk-taking [2,3], which is thought to be maladaptive in neuropathologies such as compulsive gambling and substance abuse [4,5]. Maladaptive risk assessment and increased risk-taking is more evident in adolescents [6]. This is attributed to a developmental imbalance between the slower developing cingulate and prefrontal cortices, and the more mature mesolimbic pathways [7].

Recent evidence has suggested that preconception parental stress may influence the health outcomes of their children, particularly in higher order cognitive and emotional domains. For example, children of war veterans suffering from post-traumatic stress disorder (PTSD) have been documented to display a range of cognitive impairments [8], including poor self-control behaviors (impulsivity and risk-taking) resulting in a higher prevalence of hyperactivity, aggression and delinquency [9,10]. The quality of the individual’s early home environment and the father–child relationship may be major factors contributing to the increased delinquency and inclination toward substance abuse displayed by the children of war veterans with PSTD. However, it appears increasingly possible that epigenetic inheritance may play a role in shifting offspring neurodevelopment and skewing behavioral inclinations later-in-life.

Consistent with that hypothesis, a series of preclinical studies have demonstrated that a history of paternal stress preconception shifts the behavioral phenotypes of their offspring. We previously reported that paternal corticosterone-treatment for 4 weeks prior to mating resulted in adult F1 male offspring manifesting elevated anxiety, while female offspring remained unaffected [11]. Separately, the male offspring of males subjected to maternal separation with unpredictable maternal stress (MSUS, a mouse model of early life adversity) displayed ‘reduced cautious’ behavior [12]. Three generations of MSUS offspring were found to display reduced latency to enter an open arm of the elevated-plus maze, which was interpreted as increased risk-taking behavior. However, the neural basis for this transgenerational effect remains unknown.

At a molecular level, risk-taking behavior is reported to be directly regulated by the paternally imprinted gene, growth factor receptor-bound protein 10 (Grb10). Grb10 has a complex tissue-specific imprinted expression pattern wherein only the maternal or paternal allele is expressed [13,14]. In the brain, the maternal expressed major promotor is repressed, concurrent with activation of the paternal expressed neuron-specific promoter [15]. Disruption of maternal Grb10 expression in the periphery is associated with Silver–Russell syndrome [16,17], a condition that manifests a range of metabolic and growth disruptions [18]. In the absence of paternal Grb10, mice failed to adapt by increasing their hesitancy to enter an area marked with TMT, a chemical scent mimicking fox odor [19]. It is presently unknown whether the expression of paternal Grb10 is subject to intergenerational modification and, if so, whether that would result in maladaptive behavioral responses to predator odors. It is further unknown if the expression patterns of paternal imprinted genes may be altered in offspring upon exposure of the paternal lineage to prior experiences of stress/trauma.

In the present study, we investigated the paternal influence on offspring risk assessment using the maternal separation (MS) model of early life adversity. We hypothesized that a paternal history of MS exposure would be linked to maladaptive behavioral responses of F1 PatMS offspring in the predator odor risk-assessment task (PORT), corresponding to reduced patterns of cellular activity in fear-regulating brain regions and dysregulation of paternal imprinted genes in stress-regulating brain regions, particularly in hypothalamus.

## Materials and methods

### Mice

Eight-week-old male and female C57Bl/6J mice were purchased from the Animal Resources Centre (WA, Australia) to be used as breeding pairs. All animal studies were conducted onsite at the Florey Institute of Neuroscience and Mental Health. Female mice were group-housed until mating, while males were single-housed in standard open top boxes (15 × 30 × 12cm), lined with wood-chip bedding and provided with nesting materials. Food and water were provided *ad libitum*. Mice were kept on a 12-h light/dark cycle in a holding room maintained at 45% humidity and average ambient temperature of 22°C. After at least one week of acclimatization to the animal facility, mice were randomly paired to establish breeding pairs. Males were removed after 5 days and females were left to litter down undisturbed, apart from weekly husbandry. Litters were randomly allocated to control or MS groups. Daily interventions commenced from post-natal day (PND) 3 until PND14. MS involved separating the entire nest from the dam into a separate clean cage that was thermoregulated on a heating pad to maintain a nest temperature of ∼37°C. MS was typically initiated between 1000 and 1200H, lasting for 3 h before the nest was returned to the dams in the home cage. Control litters had the cage lid removed, the entire nest lifted up and immediately replaced. Pups were weaned by 4 weeks of age and the males from each litter were group-housed (3–5 mice per cage) and kept till 8 weeks of age. A single male per litter was randomly selected and pair-mated with a naïve female to generate control and paternal MS (PatMS) F1 offspring. F1 offspring from the same litter were weaned by 4 weeks of age into standard housing cages (3–5 mice per cage), with food and water provided *ad libitum*. F1 offspring were left to age undisturbed until 12 weeks of age when behavioral testing commenced. No anaesthetics were used at any time throughout this study. All animal experiments were approved by the Florey Institute Animal Ethics Committee and conducted in accordance with the Australian code for the care and use of animals for scientific purposes as provided by the National Health and Medical Research Council of Australia.

### Predator odor risk-assessment task (PORT)

**Figure 1:**
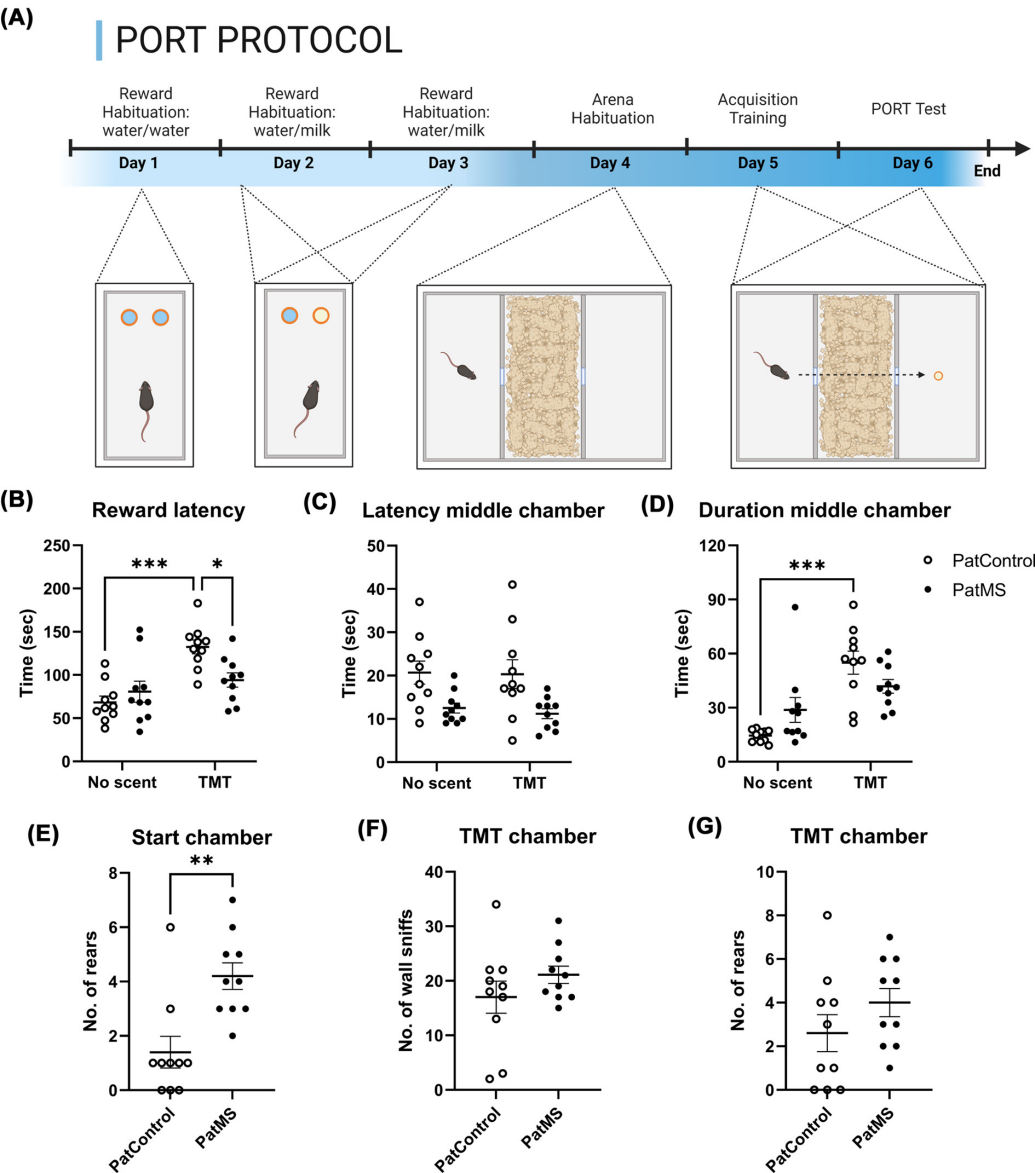
Predator odor risk-assessment task performance Representative schematic of PORT protocol (**A**). Presence of TMT did not delay reward collection of PatMS mice (**B**), which also displayed reduced latency to entering the middle chamber (**C**). TMT significantly increased middle chamber duration of control mice, but not of PatMS mice (**D**). PatMS mice recorded a greater number of rearing events in the start chamber **(E)** but wall sniffing **(F)** and rearing **(G)** in the TMT chamber were similar for both groups. Each experimental group comprises *n* = 10 mice from at least four separate litters. Data represented as mean ± SEM and analyzed by two-way ANOVA. Post-hoc *t*-test: *P<0.05, ** *P<0.01*, ***P<0.001.

The PORT protocol was adapted from Dent et al., 2020 [19] and represented in Figure 1A. Two to four mice per litter from four control and four PatMS litters were trained for PORT. Each of those F1 litters were sired by breeders from independent litters to avoid the use of half-siblings in the present study. Throughout the protocol, each home cage was restricted to 2 h of daily water access while access to standard chow remained *ad libitum*. Mice were provided 2 h of water access in their home cages after each day of training/testing. Water deprivation maintained the mice at ∼90% free-feeding bodyweight. This regime ensured that mice were sufficiently motivated to drink a 10% condensed milk reward during the initial training phase. First, mice were habituated to the milk reward over 3 days with those showing preference of >50% total fluid consumption for the reward then proceeding to PORT testing. On day 4, mice were habituated to a 3-chamber test arena (60 × 40 × 25 cm) with a 5 × 5 cm doorway, containing only unadulterated bedding in the middle chamber. The start chamber was individually randomized and was designated as whichever left or right chamber the mouse began in, while the other was designated as the third or reward chamber. On day 5, acquisition training involved five back-to-back sessions lasting a maximum of 10 min with a reward provided in the third chamber. The reward chamber contained 100 μl of 10% condensed milk in a receptacle positioned centrally on the chamber floor. Mice that failed to collect the reward within the 10-min time limit in three consecutive trials were not tested further. On day 6, the PORT test was initiated with three trials using unadulterated bedding, followed 2 h later by a trial with odor-scented bedding in the middle chamber. A transparent plastic lid was used to cover the middle chamber to limit the escape of volatile odors. Trials were limited to 10 min but ended as soon as mice began drinking the milk reward. TopScan TopView analysing system 3.0 (CleverSyst Inc, VA, U.S.A.) was used for automated tracking of the mice. The parameters tracked were duration in each chamber, latencies to enter the middle and reward chambers, and latency to collect the reward. On training days, mice were moved from the holding room to the experimental room and left for 30 min to acclimatize to the environment before procedures commenced. On test days, mice were brought to the room one cage at a time, to limit exposure to any volatile odors. A 10% ethanol spray was used to clean the entire apparatus after each mouse. To minimize potential litter effects, a maximum of three mice from an individual litter were tested. Video recordings of the test sessions were reviewed by an experimenter blinded to the experimental parameters for scoring of freezing, digging, rearing and wall-sniffing events.

### Predator odor

The predator odor used was 2,4,5-trimethylthiazoline (TMT; W332518, Sigma-Aldrich), a synthetic predator odor that is fear-inducing and elicits avoidance and anxiety-like behaviors [20]. 1% TMT solutions were prepared and stored at 4°C for up to 4 weeks before testing. TMT-scented bedding was prepared in individual sealable bags, in which 250 μl of 1% TMT was added to 200 g of bedding, equivalent to the TMT-bedding concentration previously published [19]. The bags were thoroughly mixed and prepared 2 days before the final test days, and kept in a cool dark space.

### Social dominance tube test

Social dominance tube test (SDTT) was performed in accordance with published protocols [21,22]. A separate cohort of mice consisting of 12 controls (from three separate litters) and 6 PatMS mice (from two litters) were used in this experiment. Briefly, mice were first habituated, then trained to walk through a 30 cm plexiglass tube of 3.5 cm diameter (right to left, left to right, 10 times). The next day, each mouse was pair-tested against different individuals of the opposite group. The tube was cleaned with 10% ethanol (v/v) between each trial to eliminate olfactory cues. The number of victories for individual mice was recorded and the group average was tabulated.

### RNA extraction and gene expression profiling

Mice were killed by cervical dislocation, brains removed, and hypothalamus dissected out on ice. Tissue was frozen on dry ice and stored at −80°C until later use. Tissue was disrupted using a Bioruptor Plus UCD-300 sonication device (Diagenode, Seraing, Belgium) followed by RNA extractions performed using QIAGEN QIAzol lysis protocol, in accordance to the manufacturer’s instructions. RNA concentrations and the presence of any contamination was determined using a NanoDrop 2000 spectrophotometer (Thermo Fisher Scientific, Scoresby, VIC, Australia). Presence of phenol contamination was remedied by repeating the chloroform/ethanol washes. RNA elutes were stored at −80°C until later use. 1000ng of total RNA was reverse transcribed with Superscript VILO cDNA Synthesis kit (#11754250, Invitrogen, VIC, Australia) according to the manufacturer’s instructions. Reverse transcription was performed in a Thermal Cycler Dice (TP-600, Takara Bio Inc, Kyoto, Japan), as follows: 10 min at 25°C, 60 min at 42°C, 5 min at 85°C, holding at 4°C. cDNA were diluted 1/10 then were stored at −20°C until later use.

Gene expression profiling was conducted on a ViiA7 Real-Time PCR system (#4453534, Applied Biosystems, Mulgrave VIC, Australia) with QIAGEN Quantinova SYBR green PCR kits (Catalog #208054, Clayton, VIC, Australia), in accordance with the manufacturer’s instructions. Melt curve analysis was conducted to confirm presence of a single consistent PCR product. Fold-change was calculated as 2^−∆∆Ct^ with Cyclophilin and β-actin as reference genes. Primer sequences of target genes were as follows (5′ to 3′): Grb10 forward: CACGAGTCACAACGGAGAAA, reverse CACGGGAGCACGAAGTTT; Snrpn1 forward: GAGATGCCAGACGCTTGGTTC, reverse: CAGACAGAGGATGGGTCCTGTT; PEG3 forward: GACGAGCATCGGAGGAGAAG, PEG3 reverse: GTATGACTCGGCATCCTGGG; Rtl1/PEG11 forward: CAGGCGCTAACAGTGGTTT, Rtl1/PEG11 reverse: TCAACGGTGTTGGATGAGCC; Nesp55 forward: CGAGACCGATTCTGAGACCG, Nesp55 reverse: CGCTGAGTGAGTGACTGGTT.

### c-Fos fluorescent immunohistochemistry

Separate mice that had not undergone PORT testing were used for c-Fos study of cellular activation in response to TMT exposure. Eight control mice (from five separate litters) and 4 PatMS mice (from three separate litters) were used. Briefly, mice were placed into square arenas (30 × 30 × 30 cm) that were halved with an opaque black insert partition. The floor of one half of the arena was filled with TMT-scented bedding (at least 1 cm thickness). Mice were introduced into the arena half without bedding and left for 15 min before being removed and returned to their home cages in the holding room. Ninety minutes after TMT-exposure, mice were transcardially perfused with ice-cold paraformaldehyde (4% w/v in phosphate buffered solution). Brains were removed and post-fixed for 2 h before transferring to 30% sucrose. Brains were kept overnight at 4°C or until they sunk. Brains were blotted dry and frozen into OCT media for storage at −20°C. About 30 µm coronal sections were sectioned in 1:8 series into a cryoprotectant solution composed of ethylene glycol (25%) and glycerol (25%) in PBS (pH 7.2), then stored at −20°C until subsequent use. The free floating fluorescent immunohistochemistry protocol was briefly as follows: sections were washed in PBS, blocked with 10% normal donkey serum in 5% Triton-X100/PBS for 60 min, then incubated overnight at 4°C with anti-cFos antibody (1:500, sc-166940, Santa Cruz Biotechnology) in PBS/TX (5%) − NDS (2%). Sections were then washed in PBS, then incubated for a further 90 min at room temperature in the dark with Alexa 488 donkey anti-goat secondary antibody (1:500, Invitrogen, A32814). During the final wash with mtPBS/1% Triton-X100, sections were incubated with DAPI (1:2000, Thermo Scientific, #62248) to facilitate visualization. DAKO fluorescence mounting medium (Agilent, S3023) was applied before final cover-slipping. Slides were left to dry in the dark for 24 h before proceeding to microscopy.

### Cell counting

Microscopy was performed on an Olympus BX61 and image capture was with a Hamamatsu Orca-ER digital camera (C4742-80) and ImagePro version 6.0. Sections were first visualized at 10× magnification to determine Bregma co-ordinates with referencing to *The Mouse Brain in Stereotaxic Coordinates* 2nd edition (Paxinos & Franklin, Academic Press, 2001). Regions of interest were identified, and immuno-positive cells were imaged at 20–40× magnification. Counts of c-Fos-positive cells were performed by three experimenters blinded to experimental group and individual animal identifiers. The bed nucleus of the stria terminalis (BNST) was visualized in sections spanning Bregma coordinates 0.26–0.02 mm. The anterior cingulate cortex (ACC), medial preoptic area (MPOA), anterior paraventricular thalamic nucleus (PVA) and bed nucleus of the stria terminalis medial division (BSTM) were visualized in sections spanning Bregma coordinates 0.02 to −0.46 mm. Paraventricular nuclei of the hypothalamus (PVN) were visualized in sections spanning Bregman coordinates −0.58 to −0.82 mm. The dentate gyrus (DG) and basolateral amygdala (BLA) were visualized in sections spanning Bregma coordinates −1.22 to −1.58-mm. Total cell counts were tabulated and the average count per section was calculated.

### Statistical analyses

Statistical analyses were performed using GraphPad Prism 8.0 (GraphPad Software Inc, CA, U.S.A.) or SPSS version 29. Training prior to PORT testing was analyzed with repeated-measures two-way ANOVA. Habituation to the PORT test chamber and PORT test parameters were analyzed with two-way ANOVAs, with paternal treatment and bedding as independent factors. PORT test parameters were further subject to multivariate analysis with paternal ID as the covariate factor. SDTT percentage wins per mouse was analyzed with nonparametric Mann−Whitney test and grouped outcomes were analyzed with *Χ^2^*-test. The level of significance was set at α = 0.05, except for qPCR results and cell count data that were analyzed using unpaired *t*-tests with the level of statistical significance adjusted for multiple comparisons (*P*<0.01).

## Results

### No differences during training for the predator odor risk-taking task (PORT)

Control and PatMS mice initially displayed subthreshold milk preference and most subjects exceeded the preference threshold (>50%) by the second day of training. Four control and two PatMS mice recorded <50% preference and were not tested further. Two-way RM ANOVA indicated a significant effect of day on reward preference (*F*_(1, 16)_ = 70.5, *P*<0.001) but not subject (*F*_(1, 16)_ = 1.37, *P*=0.270) or paternal treatment (*F*_(1, 16)_ = 0.975, *P*=0.338). Day × paternal treatment interaction was not significant (*F*_(1, 16)_ = 0.06665, *P*=0.801). During habituation training to the empty test arena, two-way ANOVA revealed a significant effect of chamber on duration time (*F*_(2, 51)_ = 28.7, *P*<0.001) but no significant effect of paternal treatment (*F*_(1, 51)_ < 0.001, *P*=0.978). There was also no significant chamber × paternal treatment interaction (*F*_(2, 51)_ = 0.705, *P*=0.930). Post-hoc testing showed that time spent in the middle chamber was significantly greater than the start and reward chambers, likely indicating preference for the presence of bedding over bare floors of the latter chambers.

### PatMS mice display decreased risk-assessment during the PORT

Two-way ANOVA found a significant effect of bedding condition (*F*_(1, 36)_ = 18.0, *P*<0.001) (Figure 1B) but not paternal treatment (*F*_(1, 36)_ = 2.02, *P*=0.163) on latency to reward. There was a significant bedding × paternal treatment interaction (*F*_(1, 36)_ = 7.67, *P*=0.009). The presence of TMT resulted in significantly increased reward latencies for control mice (no scent vs. TMT; *P*<0.001). This effect was not observed for PatMS mice (*P*=0.887). With exposure to TMT, PatMS mice recorded significantly reduced reward latencies compared with control mice (*P*<0.05). PORT test results were further subject to ANCOVA analysis to determine if paternal ID was a significant covariable. Offspring reward latency was not significantly affected by paternal ID (*F*_(1,39)_ = 1.43, *P*=0.240).

There was a significant effect of paternal treatment on the latency to middle chamber (*F*_(1, 36)_ = 14.4, *P*<0.001) (Figure 1C). There was no significant effect of bedding condition (*F*_(1, 36)_ = 0.139, *P*=0.712) nor a bedding × paternal treatment interaction (*F*_(1, 36)_ = 0.0389, *P*=0.845). ANCOVA analysis indicated that latency to middle chamber was not significantly affected by paternal ID (*F*_(1,39)_ = 0.371, *P*=0.546). The total duration spent in the start chamber was not affected by paternal treatment (*F*_(1, 36)_ = 0.307, *P*=0.583), but there was a significant bedding effect (*F*_(1, 36)_ = 7.12, *P*=0.011). The bedding × paternal treatment interaction was not significant (*F*_(1, 36)_ = 0.00169, *P*=0.967). There was a significant effect of bedding on total duration in the middle chamber (*F*_(1, 36)_ = 27.3, *P*<0.001) (Figure 1D) but no effect of paternal treatment (*F*_(1, 36)_ = 0.0122, *P*=0.913). ANCOVA analysis showed that the total duration in the middle chamber was not significantly affected by paternal ID (*F*_(1,39)_ < 0.001, *P*=0.988). Bedding × paternal treatment interaction was significant (*F*_(1, 36)_ = 7.14, *P*=0.011). The presence of TMT significantly increased middle chamber duration of control mice (no scent vs. TMT; *P*<0.001), but this effect was not observed for PatMS mice (*P*=0.390). There was no significant difference in middle chamber duration of control and PatMS mice when TMT was present (*P*=0.388).

Total distance moved was neither affected by bedding (*F*_(1, 36)_ = 1.14, *P*=0.261) nor paternal treatment (*F*_(1, 36)_ = 2.88, *P*=0.12), and the bedding × paternal treatment interaction was not significant (*F*_(1, 36)_ = 0.05, *P*=0.827). These indicate no locomotion-related effects are likely contributing to the PORT differences.

Different types of exploratory actions displayed by the mice during the TMT-test sessions were annotated. There was an absence of digging and freezing events, both common anxiety- and stress-associated actions [23,24]. Instead, thigmotaxis (rearing and sniffing along the walls) was observed [25,26]—actions associated with the search and location of a potential predator [27,28]. Quantification of those displays revealed that PatMS mice were rearing significantly more in the start chamber (*P*=0.002; Figure 1E). Bouts of wall-sniffing (Figure 1F) and rearing in the TMT-scented chamber (Figure 1G) did not significantly differ between the groups.

### Differential expression of paternal imprinted genes in hypothalamus of PatMS mice

**Figure 2:**
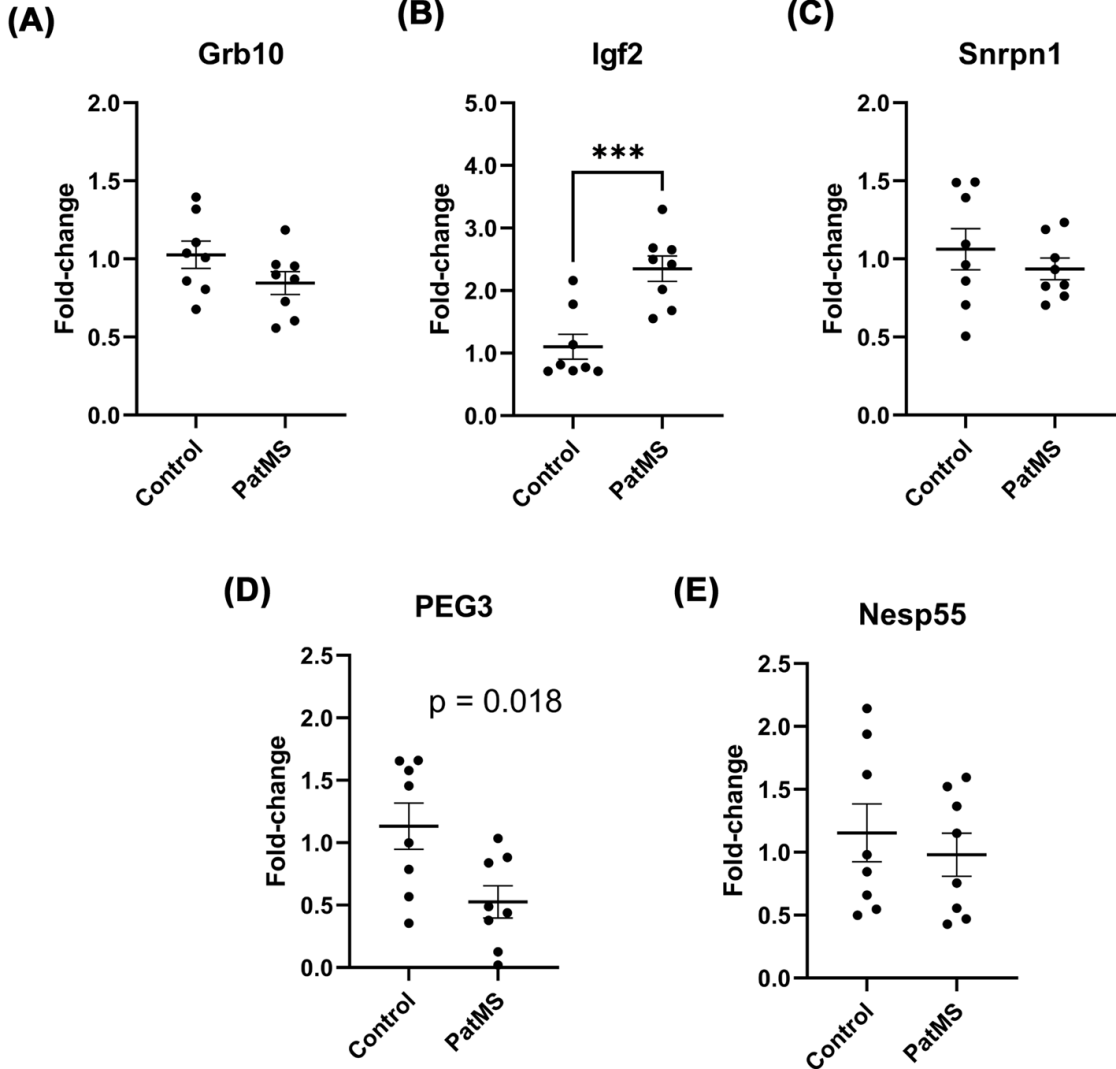
Selective impact of paternal MS exposure on paternal imprinted gene expression in male offspring hypothalamus Grb10 expression was unaffected (**A**) but Igf2 was up-regulated (**B**). Snrpn1 was also not affected (**C**) but PEG3 was significant down-regulated (**D**). There was no difference in Nesp55 expression **(E)**. Each experimental group comprises *n*=8 from at least four separate litters. Data represented as mean ± SEM and analyzed by unpaired *t*-tests; ***P<0.001.

Unexpectedly, no significant difference in hypothalamus Grb10 gene expression was detected between the groups (*t* = 1.586, *P*=0.135) (Figure 2A). This was surprising since it had been reported that paternal expression of imprinted Grb10 directly regulates risk-taking behavior in the PORT task [19], so we extended our profiling to other imprinted genes. In contrast, Igf2 levels were significantly increased in PatMS offspring (*t* = 4.381, *P*<0.001) (Figure 2B), consistent with a previous finding in a model of paternal generalized daily stress [11]. There was no difference in Snrpn1 expression between the groups (*t* = 0.852, *P*=0.409) (Figure 2C). Mean PEG3 expression was lower in PatMS mice although this did not reach statistical significance after adjusting for multiple comparisons (*t* = 2.690, *P*=0.018) (Figure 2D). We found that Rtl1/PEG11 was lowly expressed in the hypothalamus (*C*_t_ values > 35) so relative expression was not determined. Nesp55 is reportedly a molecular regulator of impulsive behavior [29], but we found that Nesp55 gene expression did not significantly differ between both groups (*t* = 0.603, *P*=0.556) (Figure 2E).

### PatMS mice display increased dominance in the social dominance tube test (SDTT)

**Figure 3:**
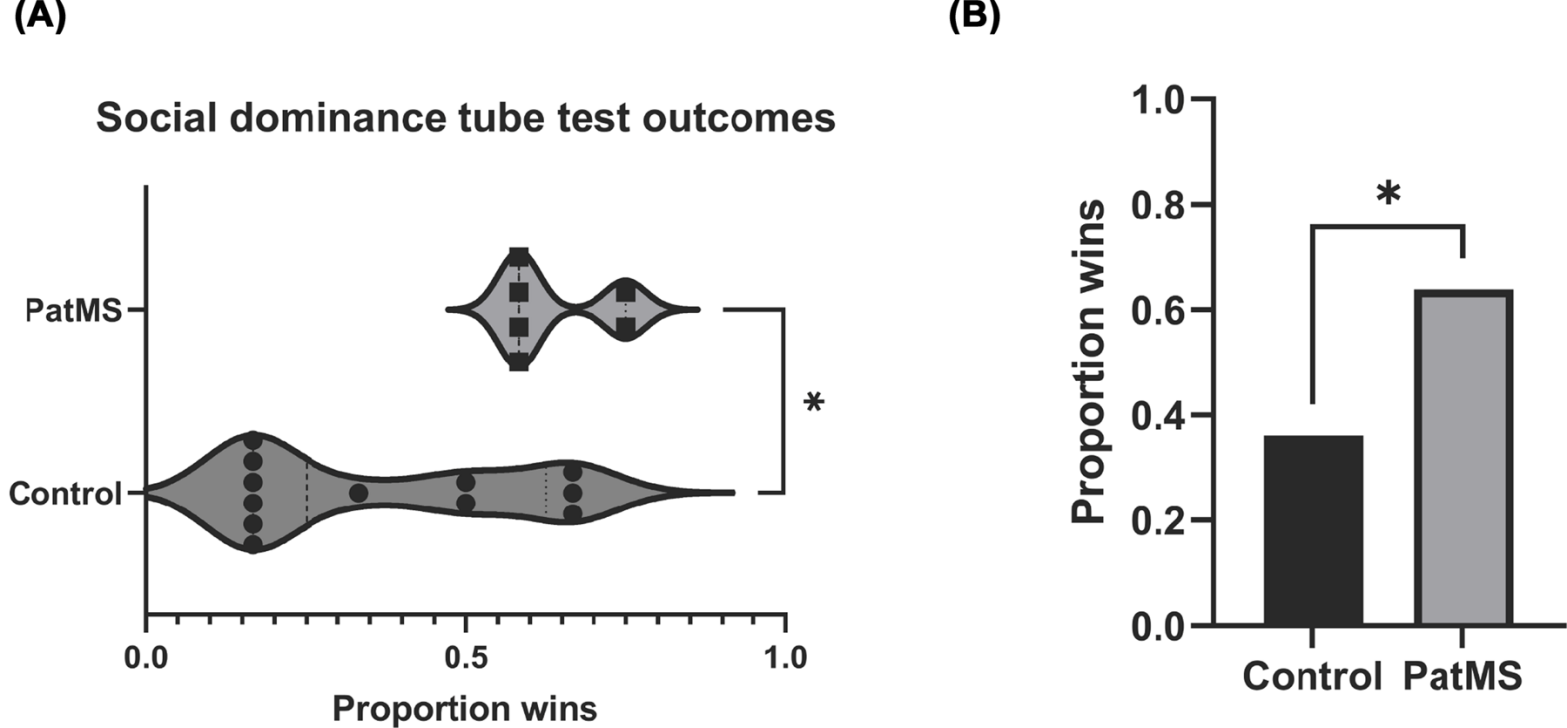
Social dominance tube test results PatMS recorded significantly more wins in the SDTT (**A**) and were overall more dominant than control mice (**B**). Data analyzed by Mann–Whitney test; *P<0.05.

The unexpected absence of altered Grb10 gene expression in PatMS mice despite differential PORT responses led us to conduct a separate assessment of behaviors linked to Grb10 (26, 27), namely social dominance. PatMS mice recorded significantly more wins than controls (*P*=0.019) (Figure 3A). Collectively, the PatMS group was more dominant than the control group (Χ^2^ df = 1, *N*=72) = 5.556, *P*=0.0184) (Figure 3B). This result, consistent with increased PORT risk-taking behavior, is indicative of altered neuronal signalling processes downstream of Grb10 in PatMS mice.

### Reduced c-Fos immunoreactivity in PatMS mice following TMT exposure

**Figure 4:**
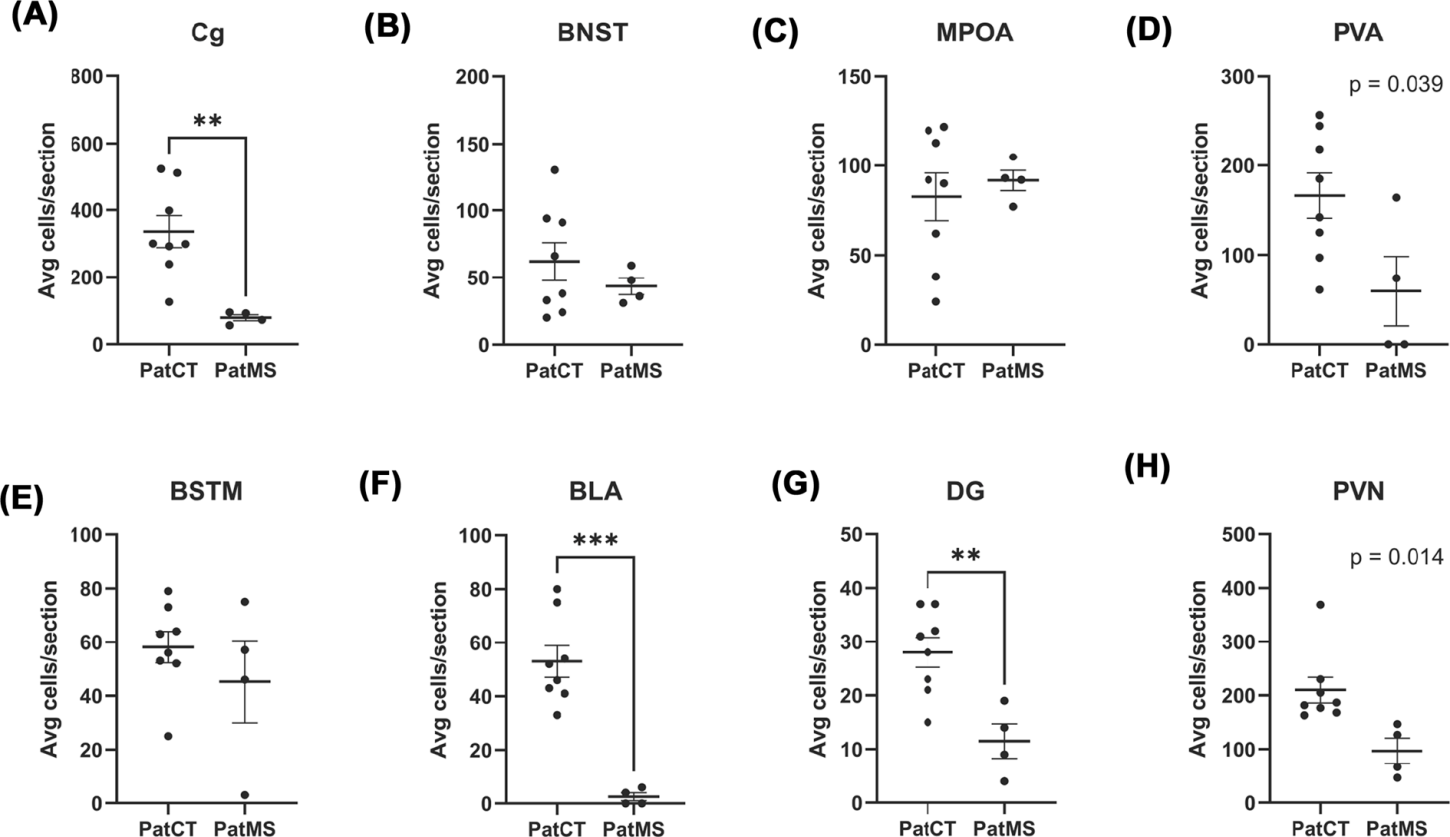
Quantification of c-Fos immunoreactivity following 1% TMT exposure c-Fos-positive cells counts were significantly lower in the Cg (**A**) of PatMS mice. There were no significant differences for the bed nucleus of the stria terminalis (BNST, **B**), medial preoptic area (MPOA, **C**), paraventricular nucleus of the thalamus (PVA, **D**), medial bed nucleus of the stria terminalis (BSTM, **E**). PatMS brains also had significantly fewer c-Fos-positive cells in the basolateral amygdala (BLA, **F**) and dentate gyrus of the hippocampus (DG, **G**). Strong trend for reduced number of c-Fos positive cells in the paraventricular nuclei of the hypothalamus (PVN, **H**) of PatMS mice. Each experimental group comprises mice pooled from three to four litters. Data presented as mean ± SEM and analyzed by unpaired *t*-tests with adjusted statistical thresholds to account for multiple *t*-tests; **P<0.01, ***P<0.001.

c-Fos mapping was used to identify specific regions where dysregulation of neural responses to TMT exposure was evident in PatMS mice. After accounting for multiple comparisons, there were significantly fewer c-Fos positive cells in the cingulate cortex (Cg) (*P*=0.004, Figure 4A) of PatMS mice, compared with control mice. No significant differences in c-Fos positive cell numbers were detected for the BNST (*P*=0.396, Figure 4B), MPOA (*P*=0.652, Figure 4C), PVA (*P*=0.039, Figure 4D) or BSTM (*P*=0.355, Figure 4E). There were significantly fewer numbers of c-Fos positive cells in the BLA (*P*<0.001, Figure 4F) and dentate gyrus (*P*=0.005, Figure 4G). Mean cell count was lower in the PVN for PatMS mice compared with controls but this was not statistically significant after adjusting for multiple comparisons (*P*=0.014, Figure 4H). In addition to those regions, c-Fos immunoreactivity was observed in the piriform cortex, dorsal and ventral endopiriform nuclei, and suprachiasmatic nucleus (data not shown).

**Figure 5:**
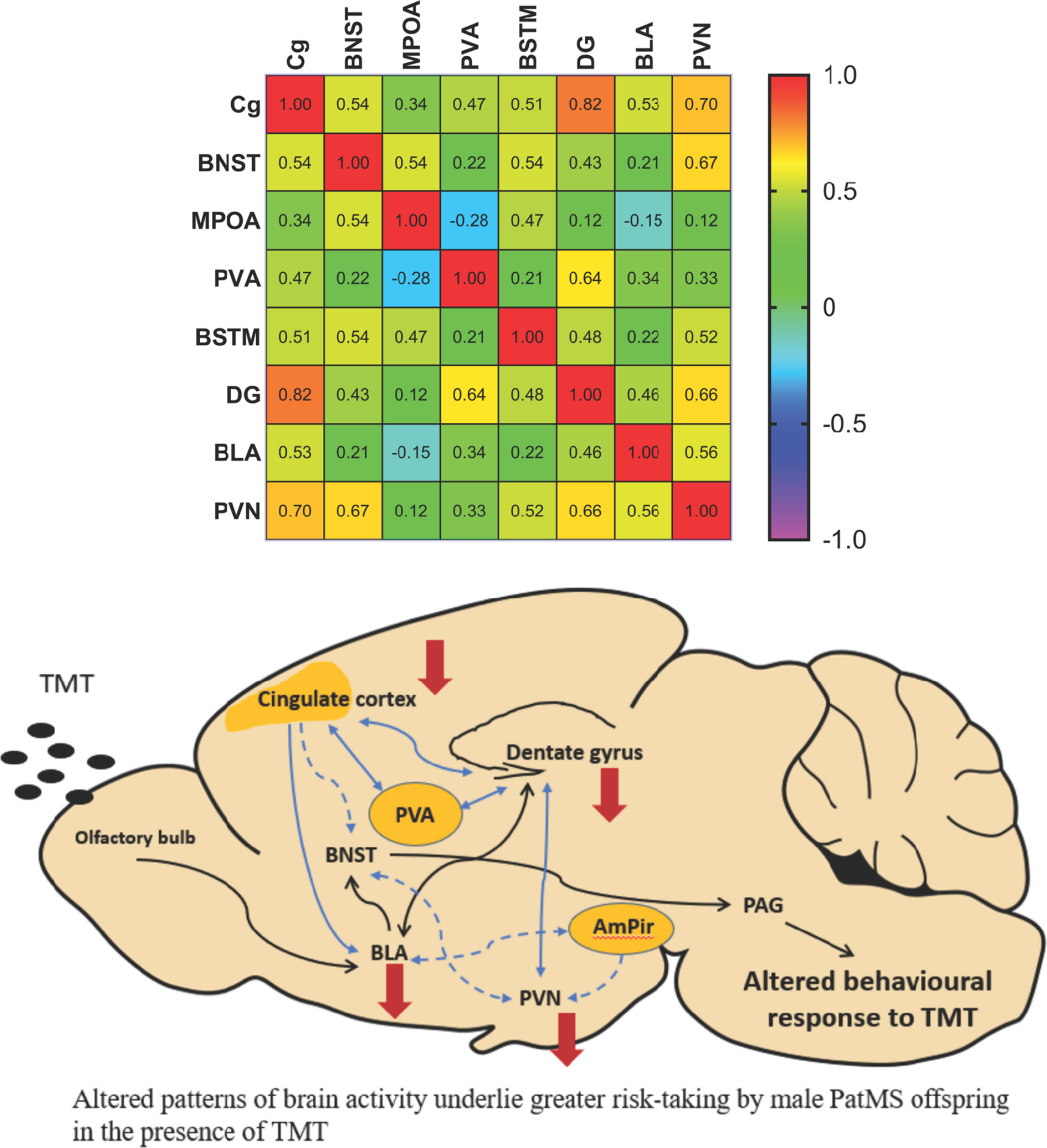
Correlation analyses reflect functional connectivity and reveal the network that underlies alter TMT responses Correlation matrix indicating significant correlations between c-Fos activity in the Cg with the DG and PVN. There were also significant positive corrections between the DG with the PVA and PVN. We propose that increased risk-taking behavior of PatMS mice observed in PORT results from dampened neuronal responses in key brain regions that regulate TMT responses, with the cingulate cortex serving as a key modulator. Red arrows denote brain regions where reduced c-Fos cell counts were observed in PatMS mice after TMT exposure. Black arrows represent the current proposed neural pathway that regulates TMT-response, as identified by previous studies. Blue solid arrows denote direct inter-regional connectivity based on significant correlations identified in this study. Blue dashed lines denote the proposed indirect top-down regulation of PVN output by the Cg. Data analyzed by Pearson’s correlation analysis and Pearson *r*-values are indicated in the heat grid. The corresponding statistical results are provided in Supplementary Table S1. Schematic designed with adapted TMT-response circuitry information from Rosen et al., 2015 [49] and Kondoh et al., 2016 [50].

Pearson’s analysis was used to construct a correlation matrix that reflected functional connectivity [30–32]. This revealed significant correlations of c-Fos activity between the Cg and the DG (*P*=0.001) and the PVN (*P*=0.012) (Figure 5, see Supplementary Table S1 for full statistical results). There were also significant relationships between c-Fos activity in the DG with the PVA (*P*=0.026) and PVN (*P*=0.020), and between BNST and PVN (*P* =0.018). These results suggest that dampened neuronal responses within the Cg, DG and BLA contribute to the abnormal behavioral responses of PatMS to TMT exposure, with the Cg being the primary modulatory hub (see proposed schematic in Figure 5).

## Discussion

Our study demonstrates a potential link between a paternal history of stress/trauma and the proclivity for their offspring to engage in risky behaviors, having found altered behavioral and cellular responses to predator scent in male PatMS offspring. This adds to an increasing body of evidence that paternal stress preconception impacts the behavioral and physiological stress responses of offspring. We also found reduced cellular activity in fear-regulating brain regions in PatMS mice, suggesting the presence of a broader dysfunction to the neural circuitry. Interestingly, and counter to our initial hypothesis, expression of the paternally imprinted gene Grb10 was unaltered in the hypothalamus of PatMS mice. It was proposed that paternal Grb10 expression directly regulates risk-taking behavior, impulsivity and social dominance of mice [19,33]. The present study has demonstrated that those behaviors can be modified independent of Grb10 expression, likely through downstream molecular signaling pathways that remain to be elucidated.

PatMS mice displayed increased risk-taking behavior by failing to moderate their approach to the reward upon encountering TMT-scented bedding. This is the first study to directly assess risk-taking using the PORT and to demonstrate the presence of a paternal intergenerational effect. One possible explanation of this could be that the motivation of PatMS mice to claim the sweetened milk reward was greater than predator fear. However, PatMS mice recorded similar latencies to reward collection as the control group during the three ‘no scent’ trials. Furthermore, PatMS mice also recorded similar latencies to enter the middle chamber during the TMT-scented trial. Interestingly, the presence of TMT-scented bedding in the middle chamber did not delay the entry time into the middle chamber, in contrast with the observations of Dent et al [19]. We used a largely similar protocol for preparing TMT-scented bedding. One key difference could be our application of 1% TMT solution instead of 10% TMT solution by Dent and colleagues. However, we applied a larger volume such that the final TMT content was identical to the previous publication. Future validation studies will be required to confirm whether the precise concentration of the applied TMT solution affects the behavioral responses.

During the process of conducting the PORT trials, we observed mice exiting the middle chamber only to return to the starting chamber, thereby account for the increased total time spent there. The control group recorded significantly increased time spent in the middle chamber during which they exhibited sniffing and rearing behaviors, likely reflecting attempts to locate the position of a potential predator. Our observation was similar to a previous report of increased exploratory behavior in male C57Bl/6J mice that were presented with phenylethylamine (PEA, another predator odor) [25]. However, only control mice displayed increased exploration of the middle chamber exploration, further demonstrating the failure of PatMS mice to moderate their behaviors in pursuit of procuring a reward. Future studies of scent avoidance and preference (e.g., TMT vs. neutral odors vs. female estrous urine) would still be necessary to confirm whether PatMS offspring are capable of selectively moderating their behaviors in the presence of a range of scents, or simply broadly impulsive. It would also be interesting to determine if other cognitive domains are similarly impacted in PatMS offspring, particularly in relation to contextual conditioned fear as a direct comparison to innate fear, which this study specifically examined.

Fear-driven behaviors are displayed by various mammalian species and are crucial for survival and adapting to an uncertain environment. Dysregulation of fear processing brain regions is associated with mental disorders such as anxiety and phobias [34–36]. While the prelimbic and infralimbic regions of the ventromedial prefrontal cortex have been extensively studied in the context of conditioned fear, the brain regions that regulate innate fear responses (more specifically, to predator odor) remains poorly understood. Recent studies have found that innate fear behavioral responses (such as freezing in response to TMT) involve the anterior cingulate cortex and its inhibitory control of BLA outputs [37]. Consistent with that, we found c-Fos immunoreactivity in the cingulate cortex and BLA of all mice after presentation of TMT. However, the number of c-Fos positive cells was dramatically reduced in both regions of PatMS brains, potentially suggestive of a failure of higher order cortical behavioral regulation. In rats, TMT had also been reported to induce c-Fos mRNA in the MPOA and PVN [38]. We also observed c-Fos immunoreactivity in both regions, this confirming their involvement in TMT response. The strong trend for reduced numbers of activated PVN neurons could underpin to the failure of PatMS mice to moderate behavior in the presence of TMT since olfaction-mediated active fear responses have been proposed to be highly dependent on hypothalamic processes [39].

Given previous reports of sex-specific behavioral alterations in different mouse models of paternal stress [11,40,41], it would be important for future studies to evaluate risk-taking behaviors of female offspring to determine whether the intergenerational influence extends to females. For the present study, we only tested males in the PORT so we are unable to exclude the possibility that female PatMS offspring may also have altered PORT responses.

While this study had only studied responses to TMT (fox scent), there is evidence that the neural responses mediating rodent innate fear responses are predator-specific. A previous study of rats had found that patterns of Fos activation within the olfactory bulb, medial prefrontal cortex, striatum and medial hypothalamus differed between cat and TMT scents (principally absent in TMT) [42]. It is currently not known whether predator-specific neural responses also occur in mice and if these are also altered in PatMS offspring, so future c-Fos-mapping studies would be required to elucidate this.

A previous study of mice selectively inbred for high anxiety (HAB) reported that environmental enrichment (mimicking cognitive stimulation through exposure to novel environments) provided during early adolescence exerted sustained anxiolytic effects, and resulted in reduced c-Fos cellular expression in the BLA following TMT scent exposure [43]. It would be very interesting to determine if the post-weaning provision of environmental enrichment could moderate the intergenerational impact of maternal separation and normalize PORT behavioral responses of PatMS mice. Our group had previously reported that post-weaning environmental enrichment failed to correct the anxiety phenotype of male offspring derived from the paternal corticosterone treatment model of generalized daily stress [41]. However, it would still be interesting to explore the influence of environmental modifiers on offspring behavior in a separate rodent model of paternal stress.

Genomic imprinting is a crucial epigenetic process that influences brain function and behavior via DNA methylation-dependent regulation of gene expression. This complex process as imprinted effects may be dependent on monoallelic to parent-biased expression, which are further specific to brain region, cell types or developmental stages [44]. Dysregulation of imprinted gene expression (e.g., mutations) can lead to neurodevelopmental and neuropsychiatric disorders [45]. Grb10 is paternally imprinted in the brain and was shown to directly moderate impulsivity and risk-taking behavior in the PORT [19,33]. Since PatMS offspring had significantly altered PORT responses despite unaltered hypothalamic Grb10 gene expression, it is therefore likely that innate fear responses are driven by a wider molecular signalling network, to which Grb10 plays a small but crucial role. Further studies of the Grb10-mediated signalling cascade will be required to elucidate the molecular dysregulation that may be occurring in the brains of PatMS mice, which underlie their altered risk-taking behavioral phenotype.

In expanding our gene expression profiling studies to other paternal imprinted genes, we have provided novel evidence of the selective nature of intergenerational paternal stress effects on gene expression in the brain. A history of paternal stress exposure led to increased Igf2 gene expression in male offspring brains, which was consistent with our previous study of a glucocorticoid-supplementation model of paternal stress [11]. It was interesting to find a general decrease of PEG3 expression in PatMS mice. PEG3 deficiency promotes male-specific accelerated adiposity, diabetic-like insulin resistance and fatty liver [46]. Future studies should therefore investigate the development of metabolic syndromes in PatMS mice. Interestingly, male mice with PEG3 deficiency were reported to be of lower social rank and displayed reduced social dominance and lower win rates in the SDTT [46]. Our study found increased dominance displays despite decreased PEG3 gene expression, suggesting the presence of pro-dominance signaling pathways that do not involve PEG3. These opposing findings demonstrate the complex molecular regulation of social behavior. Further studies will be required to clarify the different patterns of brain activity that underlie the behavioral responses of dominant and insubordinate mice in the SDTT (e.g., additional c-Fos profiling studies), before subsequent studies to understand the intergenerational modifiers of that behavior can be attempted. Since imprinted genes are tightly regulated by the state of promotor DNA methylation, our findings do reflect gene-specific dysregulation of DNA methylation in F1 offspring. However, this can only be resolved through DNA methylome sequencing studies in the future. The apparent selective dysregulation of paternal imprinted genes is further demonstrated by the sparing of Snrpn1, which is associated with Prader–Willi syndrome, a condition with clinical features including behavioral problems such as impulsivity [47], which was unaffected. Rtl1/PEG11 was recently implicated in behaviors relevant to anxiety, depression, fear learning and memory, and social dominance [48], but we were unable to detect PEG11 expression in the hypothalamus at biologically relevant levels. Future studies could determine whether PEG11 is differentially expressed in other brain regions relevant to the adaptive fear response such as the locus coeruleus, thalamus and brain stem [48].

In summary, we have provided novel preclinical evidence that a preconceptual paternal history of early life trauma can modify impulsivity and risk-taking behavior of offspring. Our findings extend upon current knowledge of the neural circuitry implicated in innate fear responses. More broadly, we suggest that epigenetic inheritance may have a significant role in the increased incidence of behavioral problems, but that further research is required in order to establish whether such epigenetic inheritance in rodents also occurs in humans. Furthermore, an urgent priority in rodent models is to precisely identify the principle regulatory genes and epigenetic modifications, as well as the cellular and systems mechanisms, that mediate intergenerational epigenetic inheritance.

## Supplementary Material

Supplementary Table S1

## Data Availability

Data are available upon request.

## Open Access

Open access for this article was enabled by the participation of University Of Melbourne in an all-inclusive *Read & Publish* agreement with Portland Press and the Biochemical Society under a transformative agreement with CAUL.

## Abbreviations

ACC: anterior cingulate cortex
Cg: cingulate cortex
MS: maternal separation
PND: post-natal day
PTSD: post-traumatic stress disorder
SDTT: social dominance tube test
TMT: 2,4,5-trimethylthiazoline
