# Supplementary material for: Paternal early life stress exerts intergenerational effects on male C57Bl/6J offspring risk-taking behaviors and predator scent-induced c-Fos expression
Source: Neuronal Signal. 2023 Apr 28;7(2):NS20220097. doi: 10.1042/NS20220097 (PMC10154287; doi:10.1042/NS20220097)
Supplement: Supplementary Table S1 [file NS-2022-0097_supp.pdf]

Supplementary Data 1

p-values for Figure 5 correlation matrix

	Cg	BNST	MPOA	PVA	BSTM	DG	BLA	PVN
**Cg**		0.070	0.275	0.125	0.092	*0.001**	0.079	*0.012**
**BNST**	0.070		0.068	0.498	0.073	0.165	0.514	*0.018**
**MPOA**	0.275	0.068		0.377	0.119	0.711	0.641	0.705
**PVA**	0.125	0.498	0.377		0.505	*0.026*	0.273	0.295
**BSTM**	0.092	0.073	0.119	0.505		0.112	0.498	0.084
**DG**	*0.001**	0.165	0.711	*0.026**	0.112		0.136	*0.020**
**BLA**	0.079	0.514	0.641	0.273	0.498	0.136		0.058
**PVN**	*0.012**	*0.018**	0.705	0.295	0.084	*0.020**	0.058	
